# Mixed Culture Fermentation and Media Optimization by Response Surface Model: *Streptomyces* and *Brachybacterium* Species in Bioflocculant Production

**DOI:** 10.3390/molecules190811131

**Published:** 2014-07-29

**Authors:** Uchechukwu U. Nwodo, Anthony I. Okoh

**Affiliations:** Applied and Environmental Microbiology Research Group (AEMREG), Department of Biochemistry and Microbiology, University of Fort Hare, Private Bag X1314, Alice 5700, South Africa; E-Mail: AOkoh@ufh.ac.za

**Keywords:** bioflocculant, *Brachybacterium*, mixed culture, optimization, *Streptomyces*

## Abstract

The biofloculant production potential of a consortium of *Streptomyces* and *Brachybacterium* species were evaluated. Optimum bioflocculant yields (g/L) and flocculation activities (%) were observed for the following preferred nutritional sources: glucose (56%; 2.78 ± 0.15 g/L), (NH_4_)_2_NO_3_ (53%; 2.81 ± 0.37 g/L) and CaSO_4_·H_2_O (47%; 2.19 ± 0.13 g/L). A Plackett-Burman design revealed the critical fermentation media components. The concentrations of these components were optimized [glucose; 16.0, (NH_4_)_2_NO_3_; 0.5 and CaSO_4_·H_2_O; 1.2 (g/L)] through a central composite design with optimum bioflocculant yield of 3.02 g/L and flocculation activity of 63.7%. The regression coefficient (R^2^ = 0.6569) indicates a weak estimation of the model’s adequacy and a high lack-of-fit value (34.1%). Lack of synergy in the consortium may have been responsible for the model inadequacy observed. FTIR spectrometry showed the bioflocculant to be a heteropolysaccharide, while SEM imaging revealed an amorphous loosely arranged fluffy structure with interstial spacing of less than 1 µm.

## 1. Introduction

Biopolymeric materials, of extracellular or intracellular origin, synthesized by some species of bacteria, fungi and algae have been variously documented to mediate flocculation of suspended particles in liquid media [1,2,3,4]. These biopolymeric substances are referred to as bioflocculants. The growing interest in these biopolymers can be attributed to the advantages they possess over the conventionally used flocculants which include aluminum salts (aluminum sulphate and polyaluminum chloride), derivatives of polyacrylamide and polyethylene imines [5]. These advantages includes being innocuous and biodegradable, and thus environmentally friendly [6].

Neurodegenerative diseases such as Alzheimer’s have been associated with polyaluminum chlorides [7], while the derivatives of polyacrylamide and polyethylene imines have similarly been implicated in neurotoxicity and cancer [8,9,10]. These adverse health effects have been, among other factors, the major motivation for the search for alternative flocculants. Consequent to the aforementioned demerits, some countries in the developed economies have initiated restrictive measures aimed at curbing the application of these conventionally used flocculants in water processing [11]. 

Appreciable flocculation activities has been reported for bioflocculants produced by several prokaryotes, fungi and a few algae [12,13,14] however, the high cost of bioflocculant production and low yield has been a major limiting factor to the industrial applications of these biopolymers [15,16,17]. Hence, the continuing search for microbial species with capabilities for enhanced bioflocculant yield with high flocculation activities [18]. 

Besides bio-prospecting for novel bioflocculant-producing bacteria, strategies employed for yield optimization of microbial products include mutational analysis and manipulation of nutritional and fermentation conditions [19]. Mixed culture fermentation and the use of industrial wastes as nutritional sources are amongst other production cost reduction strategies. Furthermore, application of mathematical models including factorial and surface response (SRD) designs has proven to be advantageous towards cost minimization and yield optimization. An additional merit of identifying the contributions of the respective input variables and optimizing the proportions of identified critical input variables has been a major reason for the application of these designs [20,21,22,23]. 

In our previous studies, axenic cultures of *Brachybacterium* sp. UFH and *Streptomyces* sp. Gansen produced bioflocculants characterized as composed of uronic acids, polysaccharide, and proteins, among other components. The bioflocculants produced by the respective actinobacteria were stable to pH extremes and high temperature. Besides the optimization of yield through manipulation of fermentation conditions for the axenic cultures, further optimization process was attempted through evaluation of *Brachybacterium* sp. and *Streptomyces* sp. as a mixed culture. The critical fermentation media components were determined with the Plackett-Burman (PB) experimental model, while a central composite design (CCD) was used to optimize the identified critical media components. Application of PB and CCD was necessitated by the dearth of information on media optimization for mixed culture fermentation. The bioflocculant produced was purified and characterized.

## 2. Results and Discussion

### 2.1. The Effects of Nutritional Sources on Bioflocculant Production

The different carbon, nitrogen and cation sources evaluated for optimal utilization for bioflocculant production showed *Streptomyces* sp. and *Brachybacterium* sp. consortium to optimally utilize glucose, (NH_4_)_2_NO_3_ and CaSO_4_·H_2_O respectively (Table 1). The flocculation activities (in percentages) and bioflocculant yields (g/L) achieved by these carbon sources were: 56% and 2.78 ± 0.15 g/L (glucose), 51% and 2.52 ± 0.44 g/L (sucrose), 48% and 2.27 ± 0.18 g/L (fructose). Similarly, the nitrogen sources showed flocculation activities and bioflocculant yields of: 53% and 2.81 ± 0.37 g/L [(NH_4_)_2_NO_3_], 49% and 1.96 ± 0.21 g/L (urea), 38% and 1.99 ± 0.56 g/L respectively (Table 1). The cation sources with flocculation activities above 40% were CaSO_4_·H_2_O and MgCl_2_ (Table 1). Although glucose, [(NH_4_)_2_NO_3_] and CaSO_4_·H_2_O were the preferred nutritional sources as they respectively yielded the optimal flocculation activity, the difference in flocculation activities achieved with other nutritional components, were not statistically significant (*p* ≤ 0.05). 

**Table 1:** Nutritional sources optimally utilized by mixed culture of *Brachybacterium* sp. and *Streptomyces* sp. for the production of bioflocculant.

Carbon Source	Glucose	Lactose	Fructose	Sucrose	Maltose	Starch	
MFA (%)	56	42	48	51	33	46	
BY (g/L)	2.78 ± 0.15	2.34 ± 0.66	2.27 ± 0.18	2.52 ± 0.44	2.09 ± 0.61	1.99 ± 0.41	
Nitrogen source	Urea	(NH_4_)_2_SO_4_	(NH_4_)_2_NO_3_	(NH_4_)_2_Cl_4_	Peptone		
MFA (%)	49	36	53	38	42		
BY (g/L)	1.96 ± 0.21	2.03 ± 0.26	2.81 ± 0.37	1.99 ± 0.56	2.31 ± 0.22		
Cation source	KCl	NaCl	MgCl_2_	CaSO_4_·H_2_O	MnCl·4H_2_O	FeSO_4_	FeCl_3_
MFA (%)	31	29	41	47	32	29	37
BY (g/L)	1.58 ± 0.11	1.26 ± 0.18	1.89 ± 0.21	2.19 ± 0.13	1.74 ± 0.17	1.55 ± 0.29	1.82 ± 0.41

MFA = maximum flocculation activity; BY = Bioflocculant yield.

Nonetheless, the consortium produced bioflocculant in an amount lower than the respective axenic cultures as noted by the yields and flocculation activity. A similar trend was observed with the assessed nitrogen and cation sources. Hence, yield optimization through mixed culture fermentation is achieved only when the respective culture acts in synergy [24]. However, *Brachybacterium* sp. UFH and *Streptomyces* sp. Gansen seems to have acted in an antagonistic manner thus leading to the decline of bioflocculant yield and flocculation activity, respectively. The actinobacterial species responsible for the antagonistic effect is not known however, the decreased bioflocculant yield was taken as an indication of antagonism. 

The utilization of various nutrient sources for the production of microbial secondary metabolites have been reported for axenic cultures [3,15,25,26], including the production of bioflocculants [16,17,19]. However a dearth of information exists with respect to the use of mixed cultures in bioflocculant production, although mixed cultures effective in the degradation of environmental pollutants have been reported [27], among other applications. 

### 2.2. Critical Media Components for Bioflocculant Production

The respective nutritional sources constituting the fermentation media: glucose, (NH_4_)_2_NO_3_, CaSO_4_·H_2_O, K_2_HPO_4_ and KH_2_PO_4_ were evaluated for their respective contributions towards bioflocculant production with an experimental outlay (Table 2) in accordance with Plackett-Burman design matrix. The observed flocculation activities (measured from experimental trials) and predicted (generated through regression analysis) are in close accord (*p* ≥ 0.05). Flocculation activities of 57% and 56% were recorded at experimental trials No. 4, 6 and 11 as the optimum (Table 2). The concentrations of media components for these experimental trials were (g/L); 12.5 (glucose), 1 [NH_4_)_2_NO_3_], 0.5 (CaSO_4_·H_2_O), 5.0 (K_2_HPO_4_) and 2 (KH_2_PO_4_) for trail No. 4, 12.5 (glucose), 1 [NH_4_)_2_NO_3_], 0.3 (CaSO_4_·H_2_O), 5.0 (K_2_HPO_4_) and 2.5 (KH_2_PO_4_) for trials No. 6 and 10 (glucose), and 1 [NH_4_)_2_NO_3_], 1 (CaSO_4_·H_2_O), 5.0 (K_2_HPO_4_) and 2 (KH_2_PO_4_) for trial No. 11, respectively. In addition, the regression analysis indicates that glucose, (NH_4_)_2_NO_3_ and CaSO_4_·H_2_O had positive effects on bioflocculant production, unlike K_2_HPO_4_ and KH_2_PO_4_ (Table 3). However, the regression coefficients (R^2^); 0.5 (glucose), 0.4 [(NH_4_)_2_NO_3_] and 0.1 (CaSO_4_·H_2_O) shown by the respective nutritional sources indicated very weak impact on the production of bioflocculant by the consortium (Table 3). 

**Table 2:** The matrix of PB design for the determination of critical media components involved in bioflocculant production by *Brachybacterium* sp. and *Streptomyces* sp. Consortium.

Runs	Coded levels/Concentrations (g/L)					Flocculation Activity (%)	
	Glucose	(NH_4_)_2_NO_3_	CaSO_4_·H_2_O	K_2_HPO_4_	KH_2_PO_4_	Observed	Predicted
1	1(12.5)	1(1.5)	−1(0.3)	1(6.5)	1(2.5)	49	51.67
2	1(12.5)	−1(1.0)	1(0.5)	1(6.5)	1(2.5)	51	51.67
3	−1(10.0)	1(1.5)	1(0.5)	1(6.5)	−1(2.0)	53	52.0
4	1(12.5)	1(1.5)	1(0.5)	−1(5.0)	−1(2.0)	57	56.33
5	1(12.5)	1(1.5)	−1(0.3)	−1(5.0)	−1(2.0)	52	54.33
6	1(12.5)	−1(1.0)	−1(0.3)	−1(5.0)	1(2.5)	56	52.33
7	−1(10.0)	−1(1.0)	−1(0.3)	1(6.5)	−1(2.0)	47	48.0
8	−1(10.0)	−1(1.0)	1(0.5)	−1(5.0)	1(2.5)	49	52.67
9	−1(10.0)	1(1.5)	−1(0.3)	1(6.5)	1(2.5)	52	50.0
10	1(12.5)	−1(1.0)	1(0.5)	1(6.5)	−1(2.0)	53	51.67
11	−1(10.0)	1(1.5)	1(0.5)	−1(5.0)	1(2.5)	56	54.67
12	−1(10.0)	−1(1.0)	−1(0.3)	−1(5.0)	−1(2.0)	51	50.67

**Table 3:** Regression analysis indicating critical media components in the production of bioflocculant by *Brachybacterium* sp. and *Streptomyces* sp. Consortium.

No.	Media Components	Estimate	*t*-value	*p*-value
x1	Glucose	0.519	6.107	0.9421
x2	(NH_4_)_2_NO_3_	0.421	2.426	0.8894
x3	CaSO_4_·H_2_O	0.119	2.561	0.1527
x4	K_2_HPO_4_	−0.327	−1.336	0.3810
x5	KH_2_PO_4_	−0.244	−0.349	0.1449

The critical media components identified through the Plackett-Burman design model were glucose, (NH_4_)_2_NO_3_ and CaSO_4_·H_2_O, although their significance with respect to bioflocculant production with the consortium was low, with only glucose barely surpassing 50% while CaSO_4_·H_2_O was estimated to have about an 11% influence. Dipotassium hydrogen phosphate and potassium dihydrogen phosphate showed a negative input, respectively, on the production of bioflocculant, hence their input may be deemed as insignificant. Nonetheless, besides bioflocculant production these salts may have served in a pH buffering function thus maintaining the physiological pH of the culture balanced. The identification of critical media components serves to reduce the cost of fermentation if industrial application is envisaged. 

### 2.3. RSD Optimization of Critical Media Components for the Production of Bioflocculant

Glucose, (NH_4_)_2_NO_3_ and CaSO_4_·H_2_O were next optimized in a 3-factor-5-level central composite design (Table 4) following their emergence as critical media components in the PB design experimentation. 

**Table 4:** Central composite design matrix for critical media components showing the observed and predicted values for flocculation activity and bioflocculant yield.

Runs	Glucose	(NH_4_)_2_NO_3_	CaSO_4_·H_2_O	Flocculation Activity (%)		Bioflocculant Yield (g/L)	
				Observed	Predicted	Observed	Predicted
1	12.0(−1)	0.5(−1)	1.2(−1)	52.5	53.68	2.53	2.48
2	12.0(−1)	0.5(−1)	1.6(+1)	49.8	53.68	2.31	2.39
3	12.0(−1)	1.5(+1)	1.2(−1)	58.1	56.12	2.92	2.74
4	12.0(−1)	1.5(+1)	1.6(+1)	60.3	56.12	2.88	2.87
5	16.0(+1)	0.5(−1)	1.2(−1)	63.7	55.53	3.02	2.93
6	16.0(+1)	0.5(−1)	1.6(+1)	49.2	55.53	2.17	2.25
7	16.0(+1)	1.5(+1)	1.2(−1)	61.0	57.97	2.92	2.74
8	16.0(+1)	1.5(+1)	1.6(+1)	53.4	57.97	2.33	2.28
9	10.64(−1.73)	1.0(0)	1.4(0)	51.2	54.27	2.46	2.51
10	17.36(+1.73)	1.0(0)	1.4(0)	54.8	57.39	2.29	2.39
11	14.0(0)	0.36(−1.73)	1.4(0)	58.6	54.26	2.61	2.54
12	14.0(0)	1.74(+1.73)	1.4(0)	56.1	57.64	2.57	2.81
13	14.0(0)	1.0(0)	1.06(−1.73)	53.3	55.83	2.48	2.74
14	14.0(0)	1.0(0)	1.84(+1.73)	57.2	55.83	2.33	2.26
15	14.0(0)	1.0(0)	1.4(0)	56.4	55.83	2.31	2.44
16	14.0(0)	1.0(0)	1.4(0)	55.9	55.83	2.47	2.44
17	14.0(0)	1.0(0)	1.4(0)	56.2	55.83	2.56	2.44
18	14.0(0)	1.0(0)	1.4(0)	56.9	55.83	2.34	2.44
19	14.0(0)	1.0(0)	1.4(0)	56.0	55.83	2.71	2.44
20	14.0(0)	1.0(0)	1.4(0)	56.2	55.83	2.36	2.44

The respective proportion of critical media with the highest flocculation activities were 16.0 g/L, 0.5 g/L and 1.6 g/L of glucose, (NH_4_)_2_NO_3_ and CaSO_4_·H_2_O, respectively, following the twenty experimental trials shown in the 3-factor-5-level CCD matrix. The flocculation activity and bioflocculant yield achieved at this media components optimum were 63.7% and 3.02 g/L, respectively. 

Following analysis of variance, the response surface was fitted to a second order model (Table 5). The relatively high regression coefficient value obtained (R^2^ = 0.6569), implied a 65.69% variability with respect to enhancing bioflocculant production as earmarked by the flocculation activity shown by *Brachybacterium* sp. and *Streptomyces* sp. consortium. 

**Table 5:** Analysis of variance showing fitted quadratic polynomial model for optimization of flocculation activity by *Brachybacterium* sp. and *Streptomyces* sp. consortium fermentation.

Source	Flocculation Activity					
	DF	SS	MS	F-ratio	*p*-value	R^2^
Regression model	9	165.1615	18.3513	2.13	0.127576	0.656946
Linear	3	44.2462	14.7487	1.71	0.227695	0.175994
Quadratic	3	18.5453	6.1818	0.72	0.564227	0.073766
Lin x Lin	3	102.37	34.1233	3.96	0.042518	0.407187
Total Error	10	86.2466	8.6247			0.343054
Lack of Fit	5	85.6132	17.1226	135.18	0.000025	0.340535
Pure Error	5	0.63333	0.12667			0.002519
**Source**	**Bioflocculant Yield**					
	**DF**	**SS**	**MS**	**F-ratio**	***p*-value**	**R^2^**
Regression model	9	0.7881873	875.7637	2.43	0.091091	0.686541
Linear	3	0.3663863	0.1221288	3.39	0.061856	0.319137
Quadratic	3	0.122301	0.040767	1.13	0.382087	0.106529
Lin x Lin	3	0.2995	998.3333	2.77	0.096567	0.260876
Total Error	10	0.3598677	359.8677			0.313459
Lack of Fit	5	0.2403843	480.7687	2.01	0.230646	0.209384
Pure Error	5	0.1194833	238.9667			0.104075

DF = degree of freedom; SS = Sum of square; MS = Mean square.

On the same note, the F-test obtained from the regression analysis validates the result with probability value of 0.1276 and the coefficient for the lack-of-fit value (R^2^ = 0.341) which was not statistically significant (*p* ≤ 0.000025). Hence, there is an indication of the model adequacy for the prediction of enhanced flocculation activity following the assay conditions (Table 5).

Similarly, the analysis of variance for the bioflocculant yield (Table 5) shows a regression coefficient of R^2^ = 0.6865 thus, an indication of 68.65% adequacy. The adequacy of this model has been shown in the harvesting of high-density cultures of *Scenedesmus* sp. through flocculation [28], activity optimization for composite bioflocculant and polyaluminum chloride [29] and in bioflocculant production optimization by the axenic culture of *Halomonas* sp.V3a’ [15].

The levels of significance of the main effects of glucose, (NH_4_)_2_NO_3_ and CaSO_4_·H_2_O to the production of bioflocculant were 1.8%, 91.3% and 28.9% respectively, as indicated by the linear model (Table 6). However, following the quadratic model (NH_4_)_2_NO_3_ and CaSO_4_·H_2_O showed positive contribution to bioflocculant yield while glucose did not. The negative regression coefficients shown by glucose following the quadratic polynomial model is an indication of the low impact shown by this carbon source towards enhancing bioflocculant yield during fermentation by the consortium. The interaction between glucose, (NH_4_)_2_NO_3_ and CaSO_4_·H_2_O showed that (NH_4_)_2_NO_3_ and CaSO_4_·H_2_O was significant while the rest was not, as their coefficients of estimate were negative (Table 6).

**Table 6:** Second order polynomial model following regression analysis of flocculation activity optimization for *Brachybacterium* sp. and *Streptomyces* sp. consortium.

Parameter	Estimate	Standard Error	t-Value	*p*-Value
Intercept	−121.1504			
Glucose	18.66811	6.626398	2.82	0.018247
(NH_4_)_2_NO_3_	2.500087	22.24044	0.11	0.912721
CaSO_4_·H_2_O	63.57429	56.72536	1.12	0.288592
Glucose × Glucose	−0.2474871	0.1938682	−1.28	0.230601
(NH_4_)_2_NO_3_ × (NH_4_)_2_NO_3_	2.360935	4.069187	0.58	0.574621
CaSO_4_·H_2_O × CaSO_4_·H_2_O	3.926695	14.69922	0.27	0.794792
Glucose × (NH_4_)_2_NO_3_	−1.825	1.038307	−1.76	0.109316
Glucose × CaSO_4_·H_2_O	−6.75	2.595768	−2.60	0.026474
(NH_4_)_2_NO_3_ × CaSO_4_·H_2_O	14.75	10.38307	1.42	0.185859

**Figure 1:**
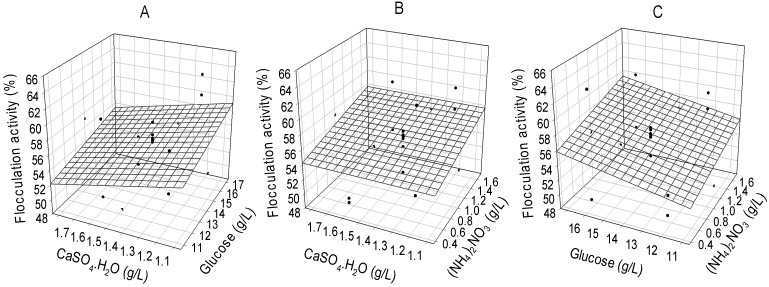
Three dimensional representations of interactions of critical media components after flocculation activity optimization following application of surface response design.

The three dimension surface response plot (Figure 1) showing the concentrations of critical media components with response (flocculation activity) revealed that at a higher concentration of glucose and lower concentration of CaSO_4_·H_2_O, flocculation activity increased (Figure 1A). Likewise, at higher concentrations of glucose and (NH_4_)_2_NO_3_ flocculation activities increased (Figure 1C) while the interaction between glucose and (NH_4_)_2_NO_3_ apparently showed no increase in flocculation activity at any level (Figure 1B). Consequently, the optimum ratio of the critical media components for the production of bioflocculant by the consortium of *Brachybacterium* sp. and *Streptomyces* sp. were: 16.0 g/L (glucose), 0.5 g/L [(NH_4_)_2_NO_3_] and 1.2 g/L (CaSO_4_·H_2_O), respectively. The maximum flocculation activity and bioflocculant yield achieved were 63.7% and 3.02 g/L, respectively. 

The feasibility of optimizing critical media components is grim if cultures are unable to grow effectively. However, since we did not ascertain the survival of the respective axenic culture in the consortium then, it will be prudent not state that growth was poor particularly as it has been shown that bioflocculant production occurs at the exponential phase of bacterial growth [5,30]. The central composite design revealed the optimum ratio of respective critical media components for bioflocculant production while the PB design showed the utmost contribution towards bioflocculant production to have come from (NH_4_)_2_NO_3_ and no clear reason can be adduced to this observation as carbon sources are known to be the most relevant factors for microbial growth. On the other hand, it may be that nitrogen sources were more important in bioflocculant production. Microbial secondary metabolites including poly-γ-glutamic acid from *Bacillus* *subtilis* RKY3 [31], bioflocculants from axenic culture of *Halomonas* sp. V3a’ [15], biosurfactants production by probiotic bacteria [32] and in the production of cold active protease by a psychrophilic bacteria belonging to the genus of Colwellia [33] have been optimized through response models. Despite the fact that surface response methodology is known for adequacy in yield optimization (secondary metabolites) and enhancing the output of desired effects, it did not show adequacy in the mixed culture fermentation as a negative regression coefficient was achieved. This may still be attributed to the antagonistic effects of the biomolecules produced in the fermentation process. 

### 2.4. Micrographic Imaging and Compositional Characteristics of the Purified Bioflocculant

**Figure 2:**
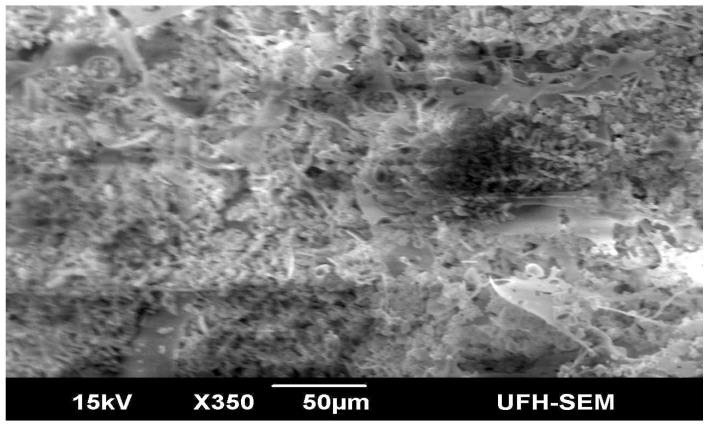
Scan electron micrographic image of the purified bioflocculant produced by the consortium of *Brachybacterium* sp. and *Streptomyces* sp.

**Figure 3:**
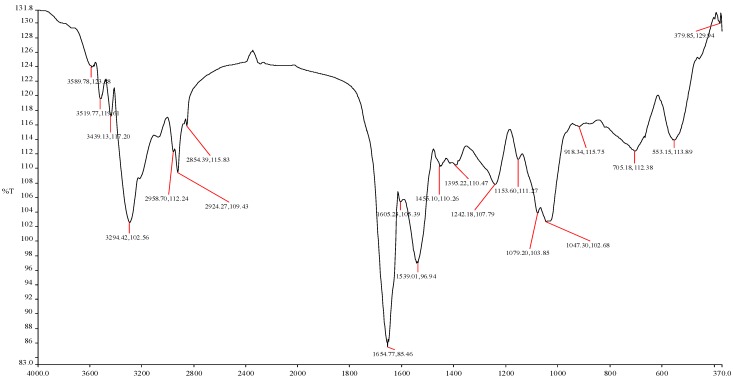
FTIR spectrum of purified bioflocculant from mixed culture fermentation of *Brachybacterium* sp. UFH and *Streptomyces* sp. Gansen.

Electron micrographic imaging of the purified bioflocculant showed loosely packed fluffy materials with irregular arrangement patterns (Figure 2). The interstices between the crispy flakes were less than 1 µm in size. The Fourier transform infrared spectrum (Figure 3) of the purified bioflocculant showed broad stretching peaks at 3589.78 to 3294.42 (cm^−1^), characteristic of hydroxyl groups from polymeric, dimeric and monomeric OH groups. Similarly, peaks from 2958.70 to 2854.39 cm^−1^ correspond to weak C–H stretching bands from methylene groups, and those from 1654.77 to 1539.01 cm^−1^ are indicative of the presence of aromatic rings [5,34,35]. Furthermore, wave numbers 1455.10 to 1395.22 cm^−1^ and 1242.18 to 1047.30 cm^−1^ shown were typical of phenol and tertiary alcohol OH bend, indicative of the presence of carboxylic groups, carboxylate ions, aromatic ring stretch and C–O and C–O–C from polysaccharides [34]. 

The loose amorphous fluffy nature of the bioflocculant is a marked variation from the clump-like nature shown by bioflocculants with high flocculation activity [5]. Similarly, the micrographic image of the respective axenic cultures was more compact, hence it may be suggested that the formation of bioflocculant was adversely affected in the consortium. The loose nature may also be understood as weak bonding between the molecules constituting the bioflocculant which leads to the low flocculation activity observed. The various functional groups such as hydroxyl, benzylic, allylic, carboxyl, esters and amino groups, amongst others, shown by FTIR spectroscopy are suggestive of an amalgam of polymers containing uronic acids, carbohydrates, glycoproteins and proteins.

## 3. Experimental Section

### 3.1. Test Bacterial Strains

Cultures of *Brachybacterium* sp. UFH (accession number HQ537131) and *Streptomyces* sp. Gansen (accession number HQ537129), preserved at −80 °C as part of the culture collection of the Applied and Environmental Microbiology Research Group (AEMREG), University of Fort Hare, South Africa were reactivated by inoculating 20 µL of the glycerol stock into a sterile 5 mL sterile broth composed of 3 g beef extract, 10 g tryptone and 5 g NaCl (per liter), respectively, and incubated overnight at 28 °C. 

### 3.2. Evaluating Carbon, Nitrogen and Cation Sources for Bioflocculant Production

The activated actinobacteria species, *Brachybacterium* sp. UFH and *Streptomyces* sp. Gansen were adjusted to cell densities of about 1.5 × 10^8^ cfu/mL and aliquots of 2 mL were inoculated into 200 mL of sterile basal salt media composed of the following (g/L): glucose, 10; tryptone, 1; K_2_HPO_4_, 5; KH_2_PO_4_, 2 and MgSO_4_·7H_2_O, 0.3. The fermentation medium was adjusted to pH 7 and incubated at a temperature of 30 °C with an agitation speed of 160 rpm for a period of 72 h. The broth, after the incubation period, was centrifuged at 3,000 rpm for 30 min at 15 °C and the cell-free supernatant was assessed for flocculation activity. Fructose, sucrose, lactose, maltose and starch respectively served as sole carbon sources, while the sole nitrogen and cation sources evaluated included urea, ammonium sulphate, ammonium nitrate, ammonium chloride, peptone, monovalent salts (KCl and NaCl), divalent salts (MgSO_4_, CaSO_4_·H_2_O, MnCl·4H_2_O, and FeSO_4_) and trivalent salts (FeCl_3_), respectively

### 3.3. Determination of Flocculation Activity

About 0.3 mL of 1% CaCl_2_ and 0.2 mL of cell free broth (bioflocculant rich broth) were added to 10 mL of kaolin suspension (4.0 g/L) in a test tube. The mixture was vortexed using a vortex mixer (VM−1000, Digisystem, New Taipei City, Taiwan) for 30 s and kept still for 5 min, after which 2 mL of the upper layer was carefully withdrawn and its optical density (OD) read spectrophotometrically (Helios Epsilon, Pittsford, NY, USA) at 550 nm wavelength. Control included repeating same process however, the bioflocculant broth was replaced with sterile (un-inoculated) fermentation medium [5,19]. All assays were in triplicates and flocculation activity calculated using the following equations:

Flocculating activity = {(A − B)/A} × 100%
(1)

where A and B are OD_550_ (optical density; 550 nm) of the control and sample, respectively.

### 3.4. Critical Media Components Determination via Plackett-Burman Design

Critical media components for the production of bioflocculant by the mixed culture were assessed using the Plackett-Burman (PB) design in an “n” variable screening of n + 1 experiments [15]. The carbon, nitrogen and cation sources yielding optimal flocculation activity were evaluated with other media components. The “n” variables were glucose, CaSO_4_·H_2_O and (NH_4_)_2_NO_3_, K_2_HPO_4_ and KH_2_PO_4_ which were investigated at two levels (concentrations) of each variable, ‘‘high” and ‘‘low” were used and was designated as +1 and −1 respectively (Table 2). All experimental trials were carried out in triplicate and the average flocculation activity was used as the response variable. Regression analysis revealed media components with significant (*p* < 0.05) effect on flocculation activity, and these components were evaluated in further optimization experiments. NCSS 2007 (Statistical Analysis and Graphics Software, Kaysville, UT, USA), was used to design and developed the PB experimental design based on the following first-order model:

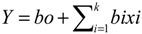
(2)
where Y = the response (flocculation activity), bo = model intercept, bi = linear coefficient, xi = level (concentrations) of the independent variable, and k = number of involved variables (media components). 

### 3.5. Critical Media Components Optimization through the Central Composite Design

Media components identified by the PB design as critical for bioflocculant production were optimized through the response surface methodology (RSM). A central composite design (CCD) model was generated and critical media components; glucose, CaSO_4_·H_2_O and (NH_4_)_2_NO_3_ were fitted into the model using the 3-factor-5-level CCD [22]. Experimental runs were all carried out in triplicate and the average of both flocculation activity and bioflocculant yield at each run were used as the response variable. The linear relationship between the response variables (flocculation activity and bioflocculant yield, respectively) and the independent variables were respectively fit to the second order polynomial model as shown below:

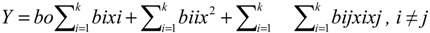
(3)
where Y = response variable (flocculation activity), bo = coefficient of interception, bi = coefficient of linear effect, bii = coefficient of the quadratic effect, bij = coefficient of interaction effect when i < j and k which are the involved variables (media components). 

### 3.6. Bioflocculant Purification

The fermentation broth was centrifuged (3,000 rpm, 30 min, 15 °C) and cell pellets separated from the supernatant by decantation. The supernatant was mixed with ice cold ethanol (95%), at volume to volume ratio of 1:4 and kept at 4 °C in a cold cabinet for 16 h. The ethanol and cell free broth mixture was centrifuged (10,000 rpm, 30 min, 15 °C) and the residue redissolved in distilled water at a ratio of 1:4 (v/v). The procedure was successively repeated twice and the purified bioflocculant was lyophilized and vacuum dried [1,23]. The lyophilized fraction was used for further studies.

### 3.7. SEM Imaging and FTIR Spectroscopy of the Purified Bioflocculant

Purified bioflocculant was placed on carbon coated stub and gold coated in a gold coating chamber, using Eiko IB.3 ION coater. Scanning electron microscopic (SEM) image of the gold coated bioflocculant was obtained using JEOL JSM-6390LV FEI XL30 (JEOL, Peabody, MA, USA) scan electron microscope. Functional groups present in the bioflocculant were determined using a Fourier transform infrared (FT-IR) spectrophotometer (2000 FTIRS Spectrometer; Perkin Elmer Systems, Waltham, MA, USA) over a wavenumber range of 4000 to 500 cm^−1^. 

## 4. Conclusions

In conclusion, the consortium of *Brachybacterium* sp. UFH and *Streptomyces* sp. Gansen produced bioflocculant with low flocculation activity and in low yield when compared to the respective axenic culture. The application of response surface design marginally improved the yield however, the model was not adequate as the antagonistic effect of the culture metabolites impeded effective synthesis of bioflocculant. Although mixed culture is an effective tool in optimization of desired effects, synergy is essential for the desired effects to be achieved. 
